# 3-[(4-Amino-5-chloro-2-ethoxy­benz­amido)meth­yl]pyrrolo[2,1-*c*][1,4]oxazin-5-ium chloride monohydrate

**DOI:** 10.1107/S160053680803986X

**Published:** 2008-12-03

**Authors:** Tai-Feng Tong, Jian Zhao, Lin Cheng, Yi-Hua Zhang

**Affiliations:** a College of Pharmacy, China Pharmaceutical University, Nanjing 210009, People’s Republic of China; b School of Chemistry and Chemical Engineering, Southeast University, Nanjing 211189, People’s Republic of China

## Abstract

The title compound, C_17_H_25_ClN_3_O_3_^+^·Cl^−^·H_2_O, is a monohydrated hydro­chloride salt of a new derivative of mosapride, which is a pharmaceutical compound possessing gastrointestinal pro-kinetic activity. The chloride anion accepts hydrogen bonds from the NH group of the pyrrolooxazine fused-ring system and from the amine group, and the water mol­ecules form hydrogen bonds that bridge between the chloride anion and the C=O bond of the amide.

## Related literature

For related structures and background information concerning mosapride, see: Kakigami *et al.* (1998 ▶); Morie *et al.* (1995 ▶); Omae *et al.* (2002 ▶); Aoki *et al.* (2007 ▶).
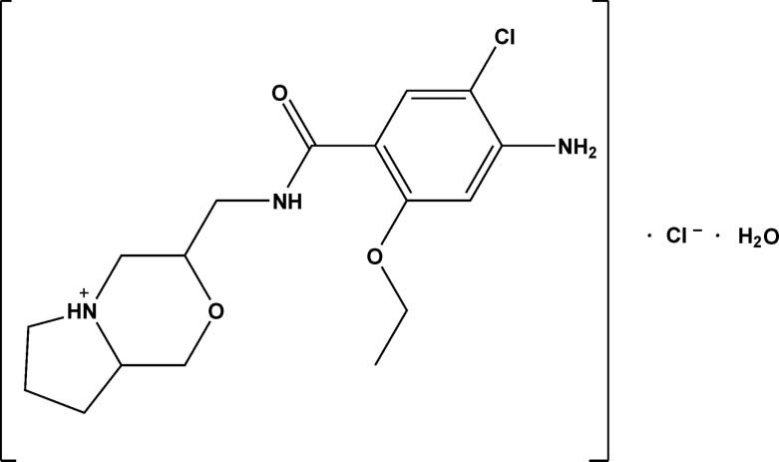


## Experimental

### 

#### Crystal data

C_17_H_25_ClN_3_O_3_^+^·Cl^−^·H_2_O
*M* *_r_* = 408.32
Orthorhombic, 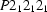

*a* = 8.0984 (9) Å
*b* = 11.1594 (13) Å
*c* = 21.843 (3) Å
*V* = 1974.0 (4) Å^3^
*Z* = 4
Mo *K*α radiation
μ = 0.36 mm^−1^
*T* = 293 (2) K
0.24 × 0.20 × 0.16 mm

#### Data collection

Bruker APEX CCD diffractometer
Absorption correction: multi-scan (*SADABS*; Bruker, 2000 ▶) *T*_min_ = 0.919, *T*_max_ = 0.945
10642 measured reflections
3848 independent reflections
3188 reflections with *I* > 2σ(*I*)
*R*_int_ = 0.037

#### Refinement

*R*[*F* ^2^ > 2σ(*F* ^2^)] = 0.050
*wR*(*F* ^2^) = 0.096
*S* = 1.06
3848 reflections
261 parameters
H atoms treated by a mixture of independent and constrained refinement
Δρ_max_ = 0.32 e Å^−3^
Δρ_min_ = −0.22 e Å^−3^
Absolute structure: Flack (1983 ▶), 1631 Friedel pairs
Flack parameter: −0.02 (7)

### 

Data collection: *SMART* (Bruker, 2000 ▶); cell refinement: *SAINT* (Bruker, 2000 ▶); data reduction: *SAINT*; program(s) used to solve structure: *SHELXS97* (Sheldrick, 2008 ▶); program(s) used to refine structure: *SHELXL97* (Sheldrick, 2008 ▶); molecular graphics: *SHELXTL* (Sheldrick, 2008 ▶); software used to prepare material for publication: *SHELXTL*.

## Supplementary Material

Crystal structure: contains datablocks I, global. DOI: 10.1107/S160053680803986X/bi2322sup1.cif

Structure factors: contains datablocks I. DOI: 10.1107/S160053680803986X/bi2322Isup2.hkl

Additional supplementary materials:  crystallographic information; 3D view; checkCIF report

## Figures and Tables

**Table 1:** Hydrogen-bond geometry (Å, °)

*D*—H⋯*A*	*D*—H	H⋯*A*	*D*⋯*A*	*D*—H⋯*A*
N1—H1*A*⋯Cl1^i^	0.84 (4)	2.66 (4)	3.384 (3)	146 (3)
N1—H1*B*⋯Cl1^ii^	0.85 (3)	2.45 (3)	3.281 (3)	169 (3)
N3—H3*C*⋯Cl1	0.92 (3)	2.18 (3)	3.077 (3)	164 (2)
O1*W*—H1*WA*⋯Cl1^iii^	0.79 (3)	2.62 (3)	3.410 (4)	176 (3)
O1*W*—H1*WB*⋯O2	0.88 (4)	1.95 (5)	2.800 (4)	161 (4)

Symmetry codes: (i) 
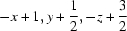
; (ii) 
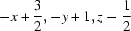
; (iii) 
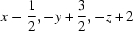
.

**Fig. 1.**
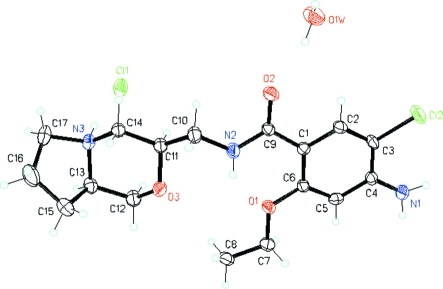
Molecular structure of the title compound with 30% displacement ellipsoids for non-H atoms.

## supplementary crystallographic information

### Comment

Mosapride citrate is a benzamide derivative that possesses a gastrointestinal prokinetic activity (Aoki *et al.* 2007). The title compound (Fig. 1) is a new mosapride derivative, and crystals of its hydrochloride salt were obtained by acidifying with hydrochloric acid then recrystallizing from methanol.

In the benzamide unit, the C=O bond distance of 1.229 (3) Å is much shorter than the C—O bond distances (1.359 (3)–1.432 (3) Å), showing it to have full double-bond character. Meanwhile, the C9—N2 distance of 1.330 (4) Å is comparable with that of C4—N1 (1.352 (4) Å), but much shorter than N2—C10 (1.441 (3) Å), N3—C13 (1.493 (4) Å), N3—C14 (1.489 (4) Å) and N3—C17 (1.492 (4) Å). Thus, the C4—N1 bond has partial double-bond character.

### Experimental

A mixture of 4-amino-5-chloro-2-ethoxybenzoic acid (8.613 g, 40 mmol), (hexahydro-1*H*-pyrrolo[2,1-*c*][1,4]oxazin-3-yl)methanamine (4.8 g, 30.72 mmol) and 1-ethyl-3-(3-dimethylaminopropyl) carbodiimide hydrochloride (11.8 g, 61.8 mmol) in CH_2_Cl_2_ (60 ml) was stirred for two hours under an argon atmosphere. The products were treated with saturated NaHCO_3_ (200 ml) and extracted with CH_2_Cl_2_ (3 × 300 ml), then the organic layer was dried with anhydrous MgSO_4_ and distilled under vacuum. The white solids were collected and dried (yield 5.856 g, 54.0%). Crystals of the title compound were obtained by acidifying with hydrochloric acid then recrystallizing from methanol.

### Refinement

With the exception of the central amide group, H atoms bonded to N and O atoms were located in difference Fourier maps and refined without restraint. Other H atoms were positioned geometrically and refined using a riding model with N—H = 0.86 Å, C—H = 0.93–0.97 Å and with *U*_iso_(H) = 1.2 times *U*_eq_(C/N).

### Crystal data

**Table:**

C_17_H_25_ClN_3_O_3_^+^·Cl^−^·H_2_O	*F*(000) = 864
*M**_r_* = 408.32	*D*_x_ = 1.374 Mg m^−^^3^
Orthorhombic, *P*2_1_2_1_2_1_	Mo *K*α radiation, λ = 0.71073 Å
Hall symbol: P 2ac 2ab	Cell parameters from 735 reflections
*a* = 8.0984 (9) Å	θ = 2.5–28.0°
*b* = 11.1594 (13) Å	µ = 0.36 mm^−^^1^
*c* = 21.843 (3) Å	*T* = 293 K
*V* = 1974.0 (4) Å^3^	Block, colourless
*Z* = 4	0.24 × 0.20 × 0.16 mm

### Data collection

**Table:**

Bruker APEX CCD diffractometer	3848 independent reflections
Radiation source: fine-focus sealed tube	3188 reflections with *I* > 2σ(*I*)
graphite	*R*_int_ = 0.037
φ and ω scans	θ_max_ = 26.0°, θ_min_ = 2.6°
Absorption correction: multi-scan (*SADABS*; Bruker, 2000)	*h* = −9→9
*T*_min_ = 0.919, *T*_max_ = 0.945	*k* = −10→13
10642 measured reflections	*l* = −26→25

### Refinement

**Table:**

Refinement on *F*^2^	Secondary atom site location: difference Fourier map
Least-squares matrix: full	Hydrogen site location: inferred from neighbouring sites
*R*[*F*^2^ > 2σ(*F*^2^)] = 0.050	H atoms treated by a mixture of independent and constrained refinement
*wR*(*F*^2^) = 0.096	*w* = 1/[σ^2^(*F*_o_^2^) + (0.0412*P*)^2^] where *P* = (*F*_o_^2^ + 2*F*_c_^2^)/3
*S* = 1.06	(Δ/σ)_max_ = 0.001
3848 reflections	Δρ_max_ = 0.32 e Å^−^^3^
261 parameters	Δρ_min_ = −0.22 e Å^−^^3^
0 restraints	Absolute structure: Flack (1983), 1631 Friedel pairs
Primary atom site location: structure-invariant direct methods	Flack parameter: −0.02 (7)

### Special details

**Table:**

Geometry. All e.s.d.'s (except the e.s.d. in the dihedral angle between two l.s. planes) are estimated using the full covariance matrix. The cell e.s.d.'s are taken into account individually in the estimation of e.s.d.'s in distances, angles and torsion angles; correlations between e.s.d.'s in cell parameters are only used when they are defined by crystal symmetry. An approximate (isotropic) treatment of cell e.s.d.'s is used for estimating e.s.d.'s involving l.s. planes.
Refinement. Refinement of *F*^2^ against ALL reflections. The weighted *R*-factor *wR* and goodness of fit *S* are based on *F*^2^, conventional *R*-factors *R* are based on *F*, with *F* set to zero for negative *F*^2^. The threshold expression of *F*^2^ > σ(*F*^2^) is used only for calculating *R*-factors(gt) *etc*. and is not relevant to the choice of reflections for refinement. *R*-factors based on *F*^2^ are statistically about twice as large as those based on *F*, and *R*- factors based on ALL data will be even larger.

### Fractional atomic coordinates and isotropic or equivalent isotropic displacement parameters (Å^2^)

**Table:**

	*x*	*y*	*z*	*U*_iso_*/*U*_eq_	
C1	0.6257 (3)	0.7564 (2)	0.77046 (11)	0.0307 (6)	
C2	0.4701 (4)	0.8084 (3)	0.76571 (13)	0.0364 (7)	
C3	0.3807 (3)	0.8034 (3)	0.71361 (13)	0.0376 (7)	
C4	0.4363 (4)	0.7438 (2)	0.66142 (12)	0.0344 (6)	
C5	0.5913 (4)	0.6900 (3)	0.66594 (13)	0.0368 (7)	
C6	0.6851 (3)	0.6961 (2)	0.71801 (12)	0.0323 (6)	
C7	0.9124 (4)	0.5928 (3)	0.66895 (12)	0.0443 (8)	
H7A	0.9261	0.6511	0.6364	0.053*	
H7B	0.8429	0.5283	0.6541	0.053*	
C8	1.0757 (4)	0.5452 (3)	0.68833 (13)	0.0455 (8)	
H8A	1.0590	0.4780	0.7150	0.068*	
H8B	1.1371	0.5201	0.6530	0.068*	
H8C	1.1360	0.6065	0.7096	0.068*	
C9	0.7127 (4)	0.7704 (2)	0.82975 (12)	0.0309 (6)	
C10	0.9629 (4)	0.7321 (3)	0.88920 (13)	0.0452 (8)	
H10A	0.8921	0.7486	0.9240	0.054*	
H10B	1.0431	0.7964	0.8860	0.054*	
C11	1.0507 (4)	0.6148 (3)	0.89873 (12)	0.0374 (7)	
H11	0.9690	0.5504	0.9021	0.045*	
C12	1.2222 (4)	0.4781 (3)	0.84947 (14)	0.0523 (10)	
H12A	1.2880	0.4646	0.8130	0.063*	
H12B	1.1348	0.4186	0.8502	0.063*	
C13	1.3298 (4)	0.4625 (3)	0.90549 (13)	0.0408 (8)	
H13	1.3596	0.3777	0.9090	0.049*	
C14	1.1561 (4)	0.6167 (3)	0.95570 (11)	0.0345 (7)	
H14A	1.2381	0.6798	0.9527	0.041*	
H14B	1.0878	0.6323	0.9913	0.041*	
C15	1.4860 (4)	0.5363 (3)	0.90920 (16)	0.0545 (9)	
H15A	1.4693	0.6151	0.8916	0.065*	
H15B	1.5759	0.4969	0.8878	0.065*	
C16	1.5225 (5)	0.5453 (4)	0.97717 (17)	0.0689 (11)	
H16A	1.6182	0.4971	0.9876	0.083*	
H16B	1.5443	0.6278	0.9886	0.083*	
C17	1.3705 (4)	0.4991 (3)	1.01002 (14)	0.0511 (9)	
H17A	1.3891	0.4191	1.0259	0.061*	
H17B	1.3406	0.5516	1.0436	0.061*	
Cl1	0.92851 (11)	0.35057 (8)	0.99188 (4)	0.0554 (3)	
Cl2	0.18901 (10)	0.87543 (9)	0.70977 (4)	0.0614 (3)	
N1	0.3486 (4)	0.7381 (3)	0.60884 (12)	0.0482 (7)	
H1A	0.266 (5)	0.780 (3)	0.5990 (16)	0.072*	
H1B	0.394 (4)	0.717 (3)	0.5755 (15)	0.072*	
N2	0.8652 (3)	0.7270 (2)	0.83413 (10)	0.0438 (7)	
H2A	0.9079	0.6941	0.8023	0.053*	
N3	1.2391 (3)	0.4983 (2)	0.96212 (11)	0.0363 (6)	
O1	0.8390 (2)	0.64810 (19)	0.72151 (8)	0.0410 (5)	
O2	0.6491 (3)	0.8222 (2)	0.87342 (8)	0.0453 (6)	
O3	1.1512 (3)	0.59417 (19)	0.84669 (9)	0.0478 (6)	
O1W	0.3524 (4)	0.8923 (3)	0.92869 (14)	0.0712 (8)	
H2	0.426 (3)	0.845 (2)	0.8005 (12)	0.038 (8)*	
H5	0.627 (3)	0.648 (3)	0.6312 (12)	0.043 (8)*	
H3C	1.161 (3)	0.443 (2)	0.9736 (11)	0.031 (8)*	
H1WA	0.368 (4)	0.954 (3)	0.9456 (15)	0.049 (12)*	
H1WB	0.444 (6)	0.887 (4)	0.9073 (18)	0.103 (16)*	

### Atomic displacement parameters (Å^2^)

**Table:**

	*U*^11^	*U*^22^	*U*^33^	*U*^12^	*U*^13^	*U*^23^
C1	0.0306 (16)	0.0293 (15)	0.0321 (14)	−0.0011 (13)	0.0022 (12)	0.0019 (12)
C2	0.0337 (18)	0.0426 (18)	0.0329 (16)	0.0061 (14)	0.0033 (13)	−0.0059 (14)
C3	0.0220 (15)	0.0445 (18)	0.0461 (17)	0.0067 (13)	−0.0004 (13)	0.0014 (15)
C4	0.0289 (15)	0.0347 (16)	0.0395 (16)	0.0000 (14)	−0.0011 (14)	0.0037 (13)
C5	0.0427 (19)	0.0369 (17)	0.0308 (15)	−0.0003 (14)	0.0093 (14)	−0.0037 (13)
C6	0.0295 (16)	0.0310 (16)	0.0364 (15)	0.0031 (13)	0.0027 (13)	0.0048 (13)
C7	0.046 (2)	0.0475 (19)	0.0396 (17)	0.0091 (16)	0.0043 (15)	−0.0063 (15)
C8	0.0380 (19)	0.0496 (19)	0.0488 (18)	0.0063 (17)	0.0112 (16)	−0.0018 (15)
C9	0.0303 (16)	0.0275 (15)	0.0350 (15)	0.0027 (12)	0.0025 (13)	0.0008 (13)
C10	0.045 (2)	0.0479 (19)	0.0430 (18)	0.0126 (16)	−0.0087 (15)	−0.0073 (15)
C11	0.0368 (16)	0.0376 (17)	0.0380 (15)	0.0030 (15)	−0.0034 (14)	−0.0028 (14)
C12	0.052 (2)	0.063 (2)	0.0426 (18)	0.0179 (19)	−0.0069 (16)	−0.0193 (17)
C13	0.0390 (19)	0.0365 (17)	0.0468 (17)	0.0072 (15)	−0.0058 (15)	−0.0087 (14)
C14	0.0361 (16)	0.0335 (16)	0.0338 (14)	0.0030 (14)	−0.0013 (13)	−0.0051 (13)
C15	0.038 (2)	0.054 (2)	0.071 (2)	0.0050 (16)	0.0032 (18)	−0.0074 (19)
C16	0.043 (2)	0.077 (3)	0.087 (3)	−0.0011 (19)	−0.016 (2)	−0.024 (2)
C17	0.059 (2)	0.0451 (19)	0.0486 (19)	0.0065 (17)	−0.0229 (17)	−0.0012 (16)
Cl1	0.0476 (5)	0.0593 (5)	0.0594 (5)	−0.0128 (4)	−0.0147 (4)	0.0070 (4)
Cl2	0.0388 (5)	0.0819 (7)	0.0635 (5)	0.0261 (5)	−0.0107 (4)	−0.0162 (5)
N1	0.0410 (17)	0.067 (2)	0.0367 (15)	0.0077 (15)	−0.0072 (13)	−0.0028 (14)
N2	0.0434 (16)	0.0529 (16)	0.0350 (13)	0.0173 (13)	−0.0074 (12)	−0.0107 (12)
N3	0.0367 (15)	0.0338 (14)	0.0385 (14)	−0.0018 (12)	−0.0079 (11)	0.0017 (12)
O1	0.0352 (12)	0.0515 (13)	0.0364 (10)	0.0138 (10)	0.0004 (9)	−0.0086 (10)
O2	0.0358 (12)	0.0588 (15)	0.0415 (11)	0.0069 (11)	0.0012 (10)	−0.0108 (11)
O3	0.0531 (14)	0.0558 (14)	0.0346 (10)	0.0229 (12)	−0.0035 (10)	−0.0039 (10)
O1W	0.0479 (18)	0.088 (2)	0.078 (2)	0.0005 (17)	0.0099 (15)	−0.0155 (18)

### Geometric parameters (Å, °)

**Table:**

C1—C2	1.391 (4)	C11—H11	0.980
C1—C6	1.413 (4)	C12—O3	1.419 (4)
C1—C9	1.483 (4)	C12—C13	1.512 (4)
C2—C3	1.350 (4)	C12—H12A	0.970
C2—H2	0.93 (3)	C12—H12B	0.970
C3—C4	1.394 (4)	C13—N3	1.493 (4)
C3—Cl2	1.750 (3)	C13—C15	1.511 (4)
C4—N1	1.352 (4)	C13—H13	0.980
C4—C5	1.395 (4)	C14—N3	1.489 (4)
C5—C6	1.369 (4)	C14—H14A	0.970
C5—H5	0.94 (3)	C14—H14B	0.970
C6—O1	1.359 (3)	C15—C16	1.517 (4)
C7—O1	1.432 (3)	C15—H15A	0.970
C7—C8	1.486 (4)	C15—H15B	0.970
C7—H7A	0.970	C16—C17	1.515 (5)
C7—H7B	0.970	C16—H16A	0.970
C8—H8A	0.960	C16—H16B	0.970
C8—H8B	0.960	C17—N3	1.492 (4)
C8—H8C	0.960	C17—H17A	0.970
C9—O2	1.229 (3)	C17—H17B	0.970
C9—N2	1.330 (4)	N1—H1A	0.84 (4)
C10—N2	1.441 (3)	N1—H1B	0.85 (3)
C10—C11	1.504 (4)	N2—H2A	0.860
C10—H10A	0.970	N3—H3C	0.92 (3)
C10—H10B	0.970	O1W—H1WA	0.79 (3)
C11—O3	1.417 (3)	O1W—H1WB	0.88 (4)
C11—C14	1.509 (4)		
			
C2—C1—C6	116.5 (3)	O3—C12—H12B	109.2
C2—C1—C9	116.8 (2)	C13—C12—H12B	109.2
C6—C1—C9	126.7 (2)	H12A—C12—H12B	107.9
C3—C2—C1	122.1 (3)	N3—C13—C15	102.8 (2)
C3—C2—H2	120.1 (17)	N3—C13—C12	110.9 (2)
C1—C2—H2	117.8 (17)	C15—C13—C12	117.6 (3)
C2—C3—C4	122.4 (3)	N3—C13—H13	108.4
C2—C3—Cl2	119.8 (2)	C15—C13—H13	108.4
C4—C3—Cl2	117.8 (2)	C12—C13—H13	108.4
N1—C4—C3	123.2 (3)	N3—C14—C11	108.7 (2)
N1—C4—C5	120.8 (3)	N3—C14—H14A	109.9
C3—C4—C5	116.0 (3)	C11—C14—H14A	109.9
C6—C5—C4	122.4 (3)	N3—C14—H14B	109.9
C6—C5—H5	122.0 (18)	C11—C14—H14B	109.9
C4—C5—H5	115.6 (18)	H14A—C14—H14B	108.3
O1—C6—C5	122.4 (2)	C13—C15—C16	104.6 (3)
O1—C6—C1	117.0 (2)	C13—C15—H15A	110.8
C5—C6—C1	120.6 (3)	C16—C15—H15A	110.8
O1—C7—C8	107.1 (2)	C13—C15—H15B	110.8
O1—C7—H7A	110.3	C16—C15—H15B	110.8
C8—C7—H7A	110.3	H15A—C15—H15B	108.9
O1—C7—H7B	110.3	C17—C16—C15	106.4 (3)
C8—C7—H7B	110.3	C17—C16—H16A	110.4
H7A—C7—H7B	108.5	C15—C16—H16A	110.4
C7—C8—H8A	109.1	C17—C16—H16B	110.4
C7—C8—H8B	109.7	C15—C16—H16B	110.4
H8A—C8—H8B	109.5	H16A—C16—H16B	108.6
C7—C8—H8C	109.7	N3—C17—C16	104.4 (3)
H8A—C8—H8C	109.5	N3—C17—H17A	110.9
H8B—C8—H8C	109.5	C16—C17—H17A	110.9
O2—C9—N2	120.3 (3)	N3—C17—H17B	110.9
O2—C9—C1	121.9 (2)	C16—C17—H17B	110.9
N2—C9—C1	117.7 (2)	H17A—C17—H17B	108.9
N2—C10—C11	109.9 (2)	C4—N1—H1A	127 (3)
N2—C10—H10A	109.7	C4—N1—H1B	121 (3)
C11—C10—H10A	109.7	H1A—N1—H1B	106 (3)
N2—C10—H10B	109.7	C9—N2—C10	123.8 (2)
C11—C10—H10B	109.7	C9—N2—H2A	118.1
H10A—C10—H10B	108.2	C10—N2—H2A	118.1
O3—C11—C10	107.6 (2)	C14—N3—C17	112.5 (2)
O3—C11—C14	109.8 (2)	C14—N3—C13	112.4 (2)
C10—C11—C14	111.7 (2)	C17—N3—C13	103.4 (2)
O3—C11—H11	109.2	C14—N3—H3C	108.4 (17)
C10—C11—H11	109.2	C17—N3—H3C	107.6 (16)
C14—C11—H11	109.2	C13—N3—H3C	112.4 (16)
O3—C12—C13	111.9 (3)	C6—O1—C7	120.4 (2)
O3—C12—H12A	109.2	C11—O3—C12	110.3 (2)
C13—C12—H12A	109.2	H1WA—O1W—H1WB	100 (4)

### Hydrogen-bond geometry (Å, °)

**Table:**

*D*—H···*A*	*D*—H	H···*A*	*D*···*A*	*D*—H···*A*
N1—H1A···Cl1^i^	0.84 (4)	2.66 (4)	3.384 (3)	146 (3)
N1—H1B···Cl1^ii^	0.85 (3)	2.45 (3)	3.281 (3)	169 (3)
N3—H3C···Cl1	0.92 (3)	2.18 (3)	3.077 (3)	164 (2)
O1W—H1WA···Cl1^iii^	0.79 (3)	2.62 (3)	3.410 (4)	176 (3)
O1W—H1WB···O2	0.88 (4)	1.95 (5)	2.800 (4)	161 (4)

Symmetry codes: (i) −*x*+1, *y*+1/2, −*z*+3/2; (ii) −*x*+3/2, −*y*+1, *z*−1/2; (iii) *x*−1/2, −*y*+3/2, −*z*+2.

## References

[bb1] Aoki, Y., Hakamata, H., Igarashi, Y., Uchida, K., Kobayashi, H., Hirayama, N., Kotani, A. & Kusu, F. (2007). *J. Chromatogr.* B**858**, 135–142. 10.1016/j.jchromb.2007.08.017 17851144

[bb2] Bruker (2000). *SMART*, *SAINT* and *SADABS* Bruker AXS Inc., Madison, Wisconsin, USA.

[bb3] Flack, H. D. (1983). *Acta Cryst.* A**39**, 876–881.

[bb4] Kakigami, T., Usui, T., Ikami, T., Tsukamoto, K., Miwa, Y., Taga, T. & Kataoka, T. (1998). *Chem. Pharm. Bull.* **46**, 1039–1043. 10.1248/cpb.46.1039 9658578

[bb5] Morie, T., Kato, S., Harada, H., Yoshida, N., Fujiwara, I. & Matsumoto, J.-I. (1995). *Chem. Pharm. Bull.* **43**, 1137–1147. 10.1248/cpb.43.1137 7586057

[bb6] Omae, T., Sakurai, M., Ashizawa, K. & Kajima, T. (2002). *Anal. Sci.* **18**, 729–730. 10.2116/analsci.18.729 12083569

[bb7] Sheldrick, G. M. (2008). *Acta Cryst.* A**64**, 112–122. 10.1107/S0108767307043930 18156677

